# Employees and the National Insurance Act

**Published:** 1912-04-06

**Authors:** 


### NATIONAL INSURANCE ACT DEPARTMENT.

### EMPLOYEES AND THE NATIONAL INSURANCE ACT.

### V.—Benefits Receivable under the Act—(*continued.*)

Maternity Benefit.

In our last two issues we dealt with the first four of the standard benefits provided by the Act—namely, medical, sanatorium, sickness, and disablement benefits. We had intended this week to set out the provisions of the Act relating to maternity benefit, and to indicate the far-reaching results which this is likely to have in the saving of life both of the mothers and of the infants. A full and lucid letter on the subject was, however, received from a solicitor, which we printed in our last issue, on page 666, under "The Editor's Letter-Box," to which we would draw particular attention. It therefore becomes unnecessary for us to deal with the maternity benefit so fully as we otherwise would.

The intention of our correspondent was to set out, in as simple a form as possible, information as to the benefit, and he referred to the difficulty women may find in disentangling the crabbed legal phrases of the Act. In this effort he has been eminently successful, but we would draw attention to three slight inaccuracies which do not affect the general description, but which, nevertheless, as we are commending the letter to the notice of our readers, it appears advisable to correct. The provisions of the Act relating to women, and particularly to married women, are so complicated and involved that it is little wonder that even a solicitor should fail to comprehend their full import and bearing upon each other, and perhaps less remarkable still when he—a man of the law—describes the complicated provisions in popular language.

The Waiting Period.

The first remark to which we would refer occurs on page 668 in the paragraph numbered 4, in which the case of a married woman, who is herself a compulsorily insured person, whilst her husband is not, is dealt with. It is there stated that in addition to the maternity benefit of 30s., which the mother will receive in her own right as an insured person, she will also receive 7s. 6d. per week sickness benefit, *though only one contribution has been paid.* The words in *italics* were inserted to emphasise the generosity of the benefit in the particular case, but they unfortunately overlook the provisions of section 8, sub-section (8), in which certain "waiting periods" are imposed before the expiration of which the benefits do not become payable.

It is stipulated in regard to maternity benefit under clause (*d*) in the case of a compulsorily insured person that he (or she) shall not be entitled to the benefit until twenty-six weeks have elapsed since his (or her) entry into insurance, and at least twenty-six weekly contributions have been paid by or in respect of him (or her). The same waiting period is imposed in the case of sickness benefit. In the case of voluntary contributors the period is extended to fifty-two weeks, and it is necessary for at least fifty-two weekly contributions to have been paid. The general object of the imposition of a preliminary period during which no benefit is receivable is to give the Act a fair start, or, in other words, to provide a fund out of which, in conjunction with the current contributions, it will be possible to pay the earliest claims as they emerge. The longer period for which maternity benefit is deferred in the case of voluntary contributors is to guard against the possibility of adverse selection against the societies. If no such provision had been made there would have been a strong inducement for a man entitled to become a voluntary contributor to take up the insurance when his wife was expecting confinement at least six months after his entry into insurance. Having received the benefit of 30s., which would more than repay his contributions, he would be under no obligation to continue his membership, and, if he decided to discontinue, he would have been a clear loss to the society.

Special Provision as to Married Women.

When a woman, who has been insured as an employed contributor, marries she has two alternative courses open to her. She may either elect to continue her contributions at the rate of 3d. per week, or she may discontinue the payment of contributions. Her rights to future benefits depend on the choice she makes, and it is a merciful provision of the Act which imposes on the society to which she belongs the necessity of furnishing her with full information as to the nature of those rights.

If she elects to continue her contributions as a voluntary contributor she will be entitled to medical benefit and sickness and disablement benefits at the reduced rates applicable, as described in the letter from "A Solicitor." The account which he gives of the benefit receivable in the event of the contributions ceasing is, however, deficient in one important qualification.

It must, of course, be understood that, having been insured for some time, a certain reserve, of greater or less amount according to the period for which she has been insured, will be held by her society on her behalf. One-third of this sum will be placed to the credit of a special fund, called the married women's suspense account, to enable her to re-enter insurance, after the death of her husband, at full benefits without any disqualification by reason of the nonpayment of contributions during her married life.

The remaining two-thirds of the sum held by her society will be applied for her benefit in providing a sum of 5s. per week on confinement for a period not exceeding four weeks on any one occasion, and in relief of sickness or distress, the latter being subject to official regulations and the discretion of her society. These benefits are, however, only to be allowed *until the fund is exhausted,* and it is further to be noted that before any payments of benefit are made so much of any sum that was credited to the approved society, as an initial reserve on her account, as may be prescribed by the Insurance Commissioners, shall be written off the amount of the reserve credited to the society.

The effect of the qualification emphasised by italics is to deal with the sum available for distribution on the same principle as applies to deposit contributors, and it will result in very meagre benefits for many years to come. For a considerable time after the commencement of insurance the reserve accumulated out of the member's own contributions will be very small, and when allowance has been made for the one-third carried to the suspense account and the deduction on account of the initial subsidy, it is clear that very little will be left from which to provide 5s. a week. It was necessary to point this out in order to prevent misunderstanding and avoid disappointment.

### Inmates of Hospitals and Maternity Benefit.

In the letter to which we have referred our readers it is stated that "when a woman, on her confinement, goes into a workhouse, public hospital, or one supported by charitable subscriptions, she will cease to receive maternity benefit, but her society must use it for the maintenance of anyone she may have dependent in part on her earnings." This is a correct statement of fact in the case where maternity benefit is the only relief obtainable, but when in addition to maternity benefit the inmate is entitled to the sickness or disablement allowance it is only the latter sum which is available for the relief or maintenance of dependants (sec. 12 (2), (ii)). In such case the sum which would otherwise be available as maternity benefit may be paid to the institution of which she is an inmate.

### Letters of Inquiry.

We are indebted to our correspondent for his extremely interesting letter, not only on the ground of the valuable matter it contained but also because it furnishes an opportunity for calling attention once again to our object in this National Insurance Act Department. We desire to assist our readers, free of charge, in all matters relating to their position under the Act, and we shall be happy to enlighten them, so far as we can, through the medium of this column if they will write us briefly on any special point of difficulty affecting themselves, enclosing with their letter the coupon to be found at the bottom of cover, p. 3.

Our description of the benefits receivable under the Act is not yet completed, but we propose next week to give an account of some of the additional or alternative benefits allowed in certain circumstances.

[Note.—Questions and Correspondence on all doubtful points are invited, and should be addressed to The Insurance Editor, The Hospital, 28 Southampton Street, Strand.]

